# Supplementary material for: Silencing RNAs expressed from W-linked PxyMasc “retrocopies” target that gene during female sex determination in Plutella xylostella
Source: Proc Natl Acad Sci U S A. 2022 Nov 7;119(46):e2206025119. doi: 10.1073/pnas.2206025119 (PMC9674220; doi:10.1073/pnas.2206025119)
Supplement: Supplementary File [file pnas.2206025119.sapp.pdf]

Supporting Information

Table S1: Summary of small RNA deep sequencing and mapping to *PxyMasc* mRNA sequence

Library	No. of reads post QC / Trimming (<= 40bp length)	No. of mapped reads	Mapped forward reads	Mapped reverse reads	Mapped reads/million total reads
**Female L1**	**3328300**	**36**	**12**	**26**	**10.81633266**
**Male L1**	**2369543**	**2**	**2**	**0**	**0.844044611**
**3h embryos**	**4814110**	**0**	**0**	**0**	**0**
**6h embryos**	**4617092**	**36**	**26**	**10**	**7.797115587**
**9h embryos**	**4284613**	**146**	**88**	**58**	**34.07542291**
**12h embryos**	**4090068**	**180**	**91**	**89**	**44.00904826**
**24h embryos**	**3741120**	**124**	**51**	**73**	**33.14515439**

Table S2: Primers used in this study.

**Primer**	**sequence**
**LA2549**	GCTCCCTTCGCGGCCTTAC
**LA4890**	CGAGTATGAGGAATTAAACAGCCTCC
**LA4891**	GAAGATATTCCGGACGTCAGTCTAG
**LA4888**	CATTAGTGGTATCTAATAGGGATCTCAACTG
**LA4889**	CGCAGTCTGCTGGTTAAGAAGAAG
**LA4886**	CTTCTTCTTAACCAGCAGACTGCG
**LA4887**	CAGCTCAAGACAAGAGTTGTTGAGAG

**LA309**	CAAGCCTCTTCGTAACAAGATCG
**LA310**	CAGCTTGATGGAGATACCACGC
**5’ RACE primer R1**	GCCACGCCGAGTAAGGCCGCGAAGGG
**5’ RACE primer R2**	CTGAATGTGCTCAGCTCAAGACAAGA
**3’ RACE primer R1**	CCCTTCGCGGCCTTACTCGGCGTGGC
**3’ RACE primer R2**	TCTCAACAACTCTTGTCTTGAGCTGAGC
**T7.PxyMasctarget.R**	GAAATTAATACGACTCACTATAGGGGAGACGAGTTT GGTTCGCCCACTG
**1948. AmCyan-RNAi-F +T7**	GAAATTAATACGACTCACTATAGGGATCTTCTCGAA GGAGGGGTCC
**1949.AmCyan-RNAi-R +T7**	GAAATTAATACGACTCACTATAGGGTATGGCCCTGT CCAACAAGTTCATC
**T7.PxyMasctarget.F**	GAAATTAATACGACTCACTATAGGGGCGGCCGCGTC TCAATTTCAC

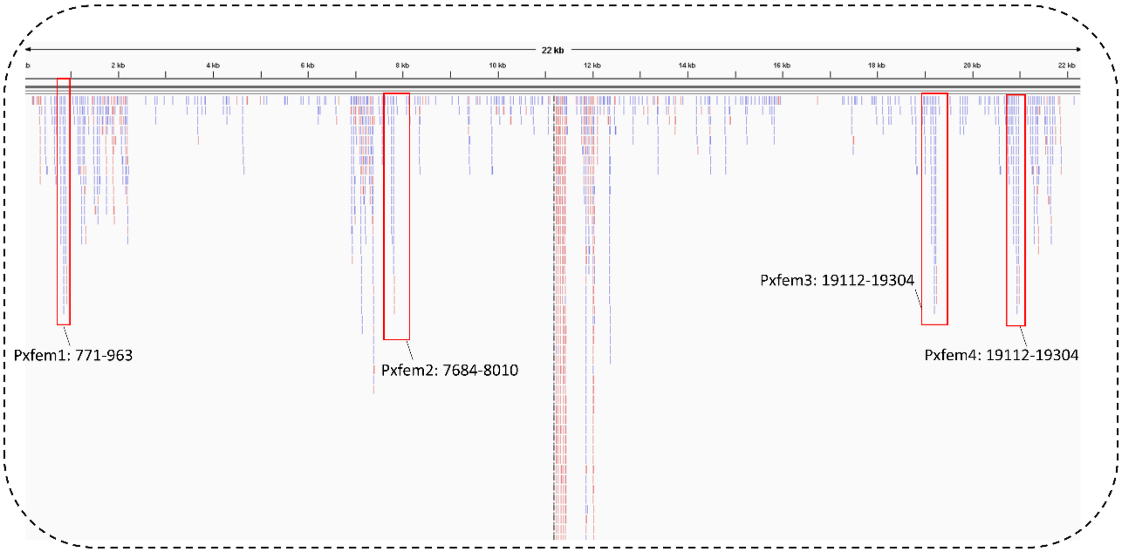

Figure S1: **Pxyfem loci occur within clusters of high ssRNA expression.** Graphic downloaded from Integrative Genomics Viewer showing reads from female L1 pool mapped to a 22Kb fragment of the CABWKK010000004 genomic scaffold encompassing the first *pxyfem* cluster (*pxyfem* 1-4). Approximate locations of the *pxyfem* loci shown. Blue read colour signifies reads mapping to reverse strand. Red read colour signifies mapping to forward strand. The four *pxyfem* loci occur with in areas with high levels of reverse strand mapping reads (i.e. producinig antisense ssRNAs against *PxyMasc* mRNA).

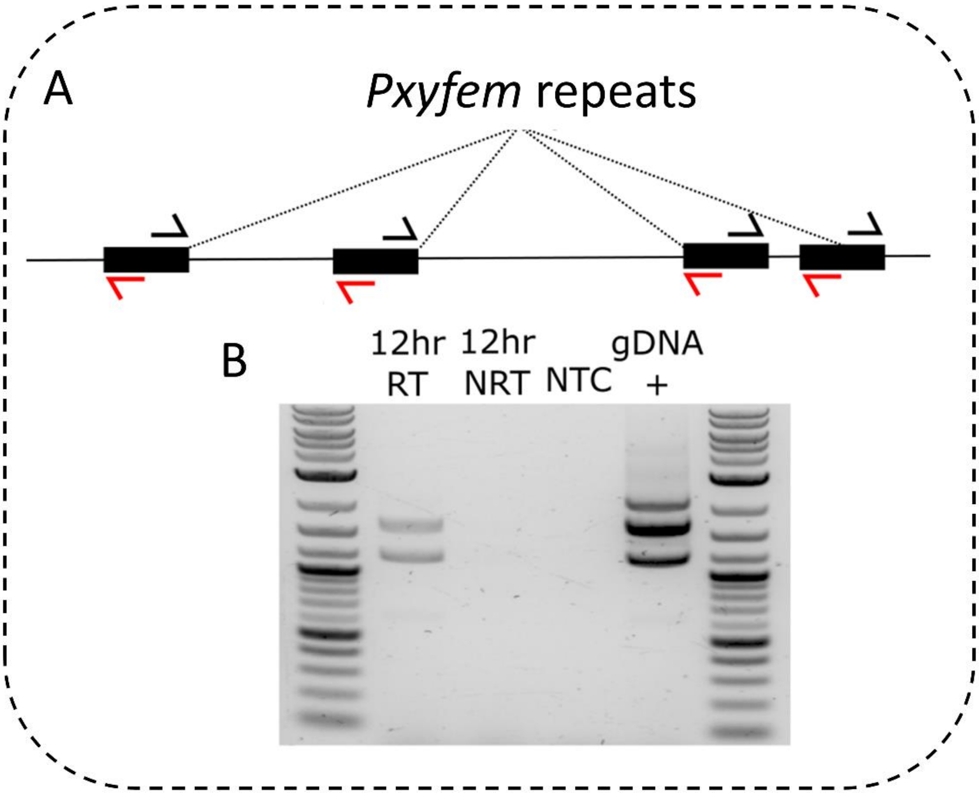

Figure S2: **Closely-linked *Pxyfem* copies are expressed on the same transcript.** A: Schematic showing four hypothetically placed *Pxyfem* copies. Primer symbols represent forward and reverse primers used in B which were conserved amongst *Pxyfem* copies. B: Results of PCR using primers shown in A. From left to right - 12h RT = RT-PCR using cDNA from 12h embryos. 12h NRT = No reverse transcriptase control of first lane. NTC = no template control for PCR in lane 4. gDNA+ = PCR using pooled female DBM gDNA. For the RT-PCR, bands would only be expected if different *Pxyfem* copies occurred on the same transcript. The presence of two bands suggests at least three *Pxyfem* copies are expressed on the same transcript.

Medium band
TTTCCTCATCTCTTGAAAAAAATAAACGAAGAAGCAGATCGCAGTCTGCTGGTTAAGAAGAT
TATGGTGAACGAAGGTAAACAGCTTCTTCTGGCGCTTGTGCCGCCAAACAAGGCGAGGGAGG
TGGATGCATTTATTGAGGGCACGCCCGTAGTGCAAAAAGAGACCCACGCCCAGTGGGCGAAC
CAGACTTGATTACCCCAGCAGTTACATGTTGGGAAGCAGAAATTTACTAGTATAATTTTCAT
TTCAGAGACTGTAACTATATCGGAAGACTATAATAATAATGCATCTAATAATGAAGAGAAAC
TTACAGAACCCGAAAAAGAAAATGAAAAAGAAGAAGTAGACTACCAAACTAATAAAATTTA
TTTATTAAGATGTAAAATATTAGGGGAAGAGTTGGAGCACAAAAAGAAGATGCATGAGGAA
GAACATTTAACTTTGTGACATTGTGCCGTGCCGTGATATAAACTAAGAACTATATAAGCACC
TGTGAACAGTTCGATGAACTTGTGATCTAACATTTAACTGTGGAACTTTGAACATTGGGCAA
TACTTACCAGTGAAGTGAACCATGAGCTACACCGTGGAGGAACTGATAGGCTTGCTAGAAGA
AAAGTCTAGACAGTTACAAAAAACACAAACAAACATAAAGAAATGTCCTAAGTCACGCCTTA
CACCTGGATACCTATCTAGCAGGTTAGAATGTATTGAAGAATTCTGGCACAACTTCAACAAA

TACCACAATACACTCGTCAAATGTGTACCGAAATCGAAGCAATCCGACATCTCTTACTTCGTG
AATGATGATTATTTTACGTGCGAAGAGATTTACATTGGCATGAAGGCTGATTTAAAAGATCT
ACTTTCATCGTGTGCGACAGACAAGACGGTACATATTTCCTCAGAATTGAGTGGCGAAAATC
AACCGCTAGTTAAGTTGTCGAGTATAAACTTGCCTACATTTGCCGGTAACTACGAAGGATGG
CAAACATTCAATGGCTTATTCCCCTCACTCGTTCATCAAAATACATCGCTATCCAATGTTCAA
AAATTACATTATTTGAAAACCAGTGTTATCGGTGAAGCAGAAGGGCTGTTGAAACATATTCA
AGAAACCGAAAGTAACTACGAGCAGGCCCTGTTGATACTTAGACAAAGATATTGAAATAAGA
GGTTAATCGTGAACTCTCTCTTCAAACGACTTTTTGGACAAAAGAAAATTGTCACGCCGACG
TCAGGGCAGTTGAGATCCCTATTAGATACCACTAATGAGTGCCTAAATAATCTGAAAAACCT
AAATGTTAGCACTAATGACTGGGGGCCGATTCTCATATTTCATGTCGTACAAAAATTGGACC
CAGATACACACAAGGCTTGGGAGGAACATGCGTACAAGGCGGACCATGATTTGCTGCCAACC
TGGGAGGAGCTTCGAACGTTCTTAGAAGGAAAATTCCGTACACTCGAACTCATCGCACCTTC
GACTTCAGCACCACGAGAGAAAAAACCTGTAAGAGAAAAAACATTCCATATCAATACACCAA
CTTCACCGACGACGACGACGAAAACCTGCATAATGTGTAGTCAGGATCATACTCTGTAGACA
ATATAGTAGGCAAAAGAAAGTCCTCTAACGC

Short band
TTGCCTACTATATTGTGAAATTGTTTGCAGTGGCACAGAGAATGATCCTGACTACACATTAT
GCAGGTTTTCGTCGTCTTCGTCGGTGAAGTTGGTGTATTGATATGGAATGTTTTTTCTCTTA
CAGGTTTTTTCTCTCGTGGTGCTGAAGTCGAAGGTGCGATGAGTTCGAGTGTACGGAATTTT
CCTTCTAAGAACGTTCGAAGCTCCTCCCAGGTTGGCAGCAAATCATGGTCCGCCTTGTACGCA
TGTTCCTCCCAAGCCTTGTGTGTATCTGGGTCCAATTTTTGTACGACATGAAATATGAGAAT
CGGCCCCCAGTCATTAGTGCTAACATTTACGTTTTTCAAATTATTTACGCACTCATTAGTGGT
ATCTAATACGGATCTCTACTGCCCTGACGTCGGCGTGACAATTTTCTTTTGTCCAAAAAGTCG
TTTGAAGATAGAGTTAAATTTCCCATTCAATTCCTAGTCGACTGTAGATGCTTACTCTCTGT
ATGATTCCGTCTAGCGCTATTAGGAAGAGCAAGGGCGACAAAAGACAGCCCTGACGGATCCC
AGCGTGCACGGCGACATCCTCCGAGATTAGGCCAATGAGAGATGGCCTTTATAAGATGTATT
ATCTTAATCGGGACACCAACGGATAATAAGCGGGACCACAAGCTGCACCATCTCAACGTATC
GAAAGCTTTTTCGAAATCGACGAAAGTAAGGTATACCTCCCTCTGCCATTCTGATGCTTGTTC
AAGTATGATACGGAGAGTGTTAATTTGGTAGGTGCAAGAGCGATTAAGGCGAAAGCTTTTTA
TTTTCTTTTTCGGGTTCTGTAAGTTTCTCTTCATTATTATTAATGTCTTCTGATATAGTTACA
GTCTCTGAAATGAAAATTATACTAGTAAATCCAGCTTTATATATTATAATTTCTGCTTCCCA
ACATGTAACTGCTGGGGTCAAGTTTGGTTCGCCCACTGGGCGTGGGTCTCTTTTTGCACTACG
GGCGTGCCCTCAATAAATGCATCCACCTCCCTCGCCTTGTTTGGCGGCACAAGCGCCAGAAGA
AGCTGTTTCCTCGTCACATAATCTTCCGACTTCTTCTTAACCAGCAGACTGCGATCTGCTTCT
TCGTTTATT

Large band
CTCTTGACAAACAATAAACGAAGAAGCAGATCGCAGTCTGCTGGTTAAGAAGAAGTCTGCTG
ATTAAGCGAGCCCTGTTGATCCTTATACGAAGATAGTGAAATAAAAGGTTGATCGGGAACTC
TCTCTTCTTACGACTTTTTGGACAAAAGAGAATTGTCACGCCGACGTCCGGGGAGTTGACAT
CCCTATTACATACCACTAATGAGTGCCTAAATAATTTGAATAATCTAAATGTTAGCACTAAT
GACTGTGGGGCCGATTCTCATATTTCATGTCGTACAAAAATTGGACCCAGATACACACAAGG
CTATACTATACCGAAGATATTCCGGACGTCAGTCTACACCAGATTAGTATGGGTCTCAAACA
GCTAAAAAACAACAAAGCTCCGGGAGATGATGGAATAACGACGGAACTTCTGAAAGCCGGCG
GTAGACCGATTTTAATAGCACTTCGGAGGCTGTTTAATTCCTCATACTCGAAGGCACAAGCC
CGGAGGCATGGAGAAGAAGTGTAGTGACTTTGTTCTTCAAGAAAGGCAACAAAGCCCTATTG
AAGAATTATAGACCCATTGCACTTCTGAGTCATGTGTACAAGCTGTTTTCGAGAGTTATTAC
GAATCGTCTCGAGCAAAGACTCGACGACTTCCAGTCACCCGAACAAGCCGGGTTCCGAAAAG

GCTATAGTACCATAGATCATATACACACGCTTCGGCAGGTTATACAGAAGACCGAGGAGTAT
AATCTACCTTTATGTCTAGCGTTTGTGGACTATGAGAAAGCCTTTGATTCTATTGAGCTCTG
GGCGATGCTTCAATCCCTTCAGCGGTGCCATATAGACTATCGCTATATCGAGGTGTTGAGAT
GTATGTACAATGCTGCCACAATGTCAGTTCGATTACACGAACATAGCACAAAACCGATCCAG
TTGCAAAGGGGCGTGAGACAGGGAGATGTTATTTCTCCGAAACTGTGCACTGGAAGATGTTT
TTAAGCTTGTAGAGTGGAAAAGACTGGGCATTAACGTCAATGGCGAATATATCTCTCATCTA
CGATTTGCTGATGACATAGTTATTATGGCGGAAACGCTGGAGGAGTTAGGCGAAATGCACAC
AGACCTCAATCATGCCTCTAAACAAGTTGGGCTGAAAATGAACATGGACAAGACAAAGGTCA
TGTCGAACGAACATGTTTCATCATCGCCCGTAACTGTAGGAGGTGTCACCATCGAAGTTGTC
GATCAGTATCCCTACCTAGGACAAGTGATCCGATTAGGTAAATCCAACTTTGATAAAGAGGT
AGCTCGTAGAATCCAACTCGGATGGGCAGCGTTCGGGAAATTACGACACATCTTCACTGAAA
ACATACCTCAGTGTCAAAAACAAAAGTTTTCAATCAGTGCGTGTTGCCAGTGATGACTTACG
AAGCCGAGACGTGGTGCTTCACCAAAGGGCTTATCCACAAGCTCAGAGTTGCTCAGCGTGCT
ATGGAAAGGGCTATGTTAGGCGTGTCCCTGCGAGATAGGATTCGTAATGAAGAAATCCGCAG
GAGAACTAAAGTTACCGACATAGCCAAAAGGATTAGCACGCTGAAGTGGCAATGGGCTGGCC
CACGTAGCCCGCAGAGCCGACGACCGCTGGAGTACAAAGGTTCTGGAGTGGAGACCCCGTGTC
GGCAAACGGCGTGTCGGTCGCCCCCCAACCCGTTGGTCTGATGATCTGCGGAAGGTAGCGGGA
AGCCGCTGGATGCAGATGGCGGGTGACCGTTTGGGGTGGCGATCGTTAGGAGAGGCCTATGT
CCAACAGTGGACTAAAGAAGGCTGAGAGAGAGAGAGACACACAAGGCTTGGGCGGACCATGA
TTTGCTGCCAACCTGGGAGGAGCTTCGAACGTTCTTAGAAGGAAAATTCCGTACACTCGAAC
TCATCGCACCTTCGACTTCAGCACCACGAGAGAACAAACCTGTAAGAGAAAAAACATTCCAT
ATCAATACACCAACTTCACCGACGACGACGACGACGAAAACCTGCATAATGTGTAGTCAGGA
TCATACTCTGTGCCACTGCAAACAATTTCACAATATAGTAGGCAAAATAAAGTCCTCTAACG
CATATGACGCCACGCCGAGTAAGGCCGCGAAAGGGA

```
#=======================================
# Aligned_sequences: 2
# 1: genomic.sequence.(b/w.pxyfem.5-6)
# 2: medium.1800bp.Band
# Matrix: EBLOSUM62
# Gap_penalty: 10.0
# Extend_penalty: 0.5
#
# Length: 1923
# Identity:    1618/1923 (84.1%)
# Similarity:  1618/1923 (84.1%)
# Gaps:         300/1923 (15.6%)
# Score: 9076.5
#=======================================

genomic            1 atttcacaatatagtaggcaaaagaaagtcctctaacgcatatgacgcca     50

medium             1 --------------------------------------------------      0

genomic           51 cgccgagtaaggccgcgaagggagccctcgccccctgcgctgaatgtgct    100

medium             1 --------------------------------------------------      0

genomic          101 cagctcaagacaagagttgttgagagtaaactactcattgaacaaacaat    150
                                                                |||.|||
medium             1 -------------------------------------------AAAAAAT      7

genomic          151 aaacgaagaagcagatcgcagtctgctggttaagaagattat-gtg-acg    198
                     |||||||||||||||||||||||||||||||||||||||||| ||| |||
medium             8 AAACGAAGAAGCAGATCGCAGTCTGCTGGTTAAGAAGATTATGGTGAACG     57

genomic          199 agg--aaacagcttcttctggcgcttgtgccgccaaacaaggcgagggag    246
```

```
                     |.|  |||||||||||||||||||||||||||||||||||||||||||||
medium            58 AAGGTAAACAGCTTCTTCTGGCGCTTGTGCCGCCAAACAAGGCGAGGGAG    107

genomic          247 gtggatgcatttattgagggcacgcccgtagtgcaaaaagagacccacgc    296
                     ||||||||||||||||||||||||||||||||||||||||||||||||||
medium           108 GTGGATGCATTTATTGAGGGCACGCCCGTAGTGCAAAAAGAGACCCACGC    157

genomic          297 ccagtgggcgaaccaaacttgattaccccagcagttacatgttgggaagc    346
                     |||||||||||||||.||||||||||||||||||||||||||||||||||
medium           158 CCAGTGGGCGAACCAGACTTGATTACCCCAGCAGTTACATGTTGGGAAGC    207

genomic          347 agaaatttactagtataattttcatttcagagactgtaactatatcggaa    396
                     ||||||||||||||||||||||||||||||||||||||||||||||||||
medium           208 AGAAATTTACTAGTATAATTTTCATTTCAGAGACTGTAACTATATCGGAA    257

genomic          397 gactataataataatgcatctaataatgaagagaaacttacagaacccga    446
                     ||||||||||||||||||||||||||||||||||||||||||||||||||
medium           258 GACTATAATAATAATGCATCTAATAATGAAGAGAAACTTACAGAACCCGA    307

genomic          447 aaaagaaaatgaaaaagaagaagtagactaccaaactaataaaatttatt    496
                     ||||||||||||||||||||||||||||||||||||||||||||||||||
medium           308 AAAAGAAAATGAAAAAGAAGAAGTAGACTACCAAACTAATAAAATTTATT    357

genomic          497 tattaagatgtaaaatattaggggaagagttggagcacaaaaagaagatg    546
                     ||||||||||||||||||||||||||||||||||||||||||||||||||
medium           358 TATTAAGATGTAAAATATTAGGGGAAGAGTTGGAGCACAAAAAGAAGATG    407

genomic          547 catgaggaagaacatttaactttgtgacattgtgccgtgccgtgatataa    596
                     ||||||||||||||||||||||||||||||||||||||||||||||||||
medium           408 CATGAGGAAGAACATTTAACTTTGTGACATTGTGCCGTGCCGTGATATAA    457

genomic          597 actaagaactatataagcacctgtgaacagttcgatgaacttgtgatcta    646
                     ||||||||||||||||||||||||||||||||||||||||||||||||||
medium           458 ACTAAGAACTATATAAGCACCTGTGAACAGTTCGATGAACTTGTGATCTA    507

genomic          647 acatttaactgtggaactttgaacattgggcaatacttaccagtgaagtg    696
                     ||||||||||||||||||||||||||||||||||||||||||||||||||
medium           508 ACATTTAACTGTGGAACTTTGAACATTGGGCAATACTTACCAGTGAAGTG    557

genomic          697 aaccatgagctacaccgtggaggaactgataggcttgctagaagaaaagt    746
                     ||||||||||||||||||||||||||||||||||||||||||||||||||
medium           558 AACCATGAGCTACACCGTGGAGGAACTGATAGGCTTGCTAGAAGAAAAGT    607

genomic          747 ctagacagttacaaaaaacacaaacaaacataaagaaatgtcctaagtca    796
                     ||||||||||||||||||||||||||||||||||||||||||||||||||
medium           608 CTAGACAGTTACAAAAAACACAAACAAACATAAAGAAATGTCCTAAGTCA    657

genomic          797 cgccttacacctggatacctatctagcaggttagaatgtattgaagaatt    846
                     ||||||||||||||||||||||||||||||||||||||||||||||||||
medium           658 CGCCTTACACCTGGATACCTATCTAGCAGGTTAGAATGTATTGAAGAATT    707

genomic          847 ctggcaaaacttcaacaaataccacaatacactcgtcaaatgtgtaccga    896
                     ||||||.|||||||||||||||||||||||||||||||||||||||||||
medium           708 CTGGCACAACTTCAACAAATACCACAATACACTCGTCAAATGTGTACCGA    757

genomic          897 aatcgaagcaatccgacatctcttacttcgtgaatgatgattattttacg    946
                     ||||||||||||||||||||||||||||||||||||||||||||||||||
medium           758 AATCGAAGCAATCCGACATCTCTTACTTCGTGAATGATGATTATTTTACG    807

genomic          947 tgcgaagagatttacattggcatgaaggctgatttaaaagatctactttc    996
                     ||||||||||||||||||||||||||||||||||||||||||||||||||
medium           808 TGCGAAGAGATTTACATTGGCATGAAGGCTGATTTAAAAGATCTACTTTC    857

genomic          997 atcgtgtgcgacagacaagacggtacatatttcctcagaattgagtggcg   1046
                     ||||||||||||||||||||||||||||||||||||||||||||||||||
medium           858 ATCGTGTGCGACAGACAAGACGGTACATATTTCCTCAGAATTGAGTGGCG    907

genomic         1047 aaaatcaaccgctagttaagttgtcgagtataaacttgcctacatttgcc   1096
```

```
                         ||||||||||||||||||||||||||||||||||||||||||||||||||
medium             908   AAAATCAACCGCTAGTTAAGTTGTCGAGTATAAACTTGCCTACATTTGCC   957

genomic           1097   ggtaactacgaaggatggcaaacattcaatggcttattcccctcactcgt   1146
                         ||||||||||||||||||||||||||||||||||||||||||||||||||
medium             958   GGTAACTACGAAGGATGGCAAACATTCAATGGCTTATTCCCCTCACTCGT   1007

genomic           1147   tcatcaaaatacatcgctatccaatgttcaaaaattacattatttgaaaa   1196
                         ||||||||||||||||||||||||||||||||||||||||||||||||||
medium            1008   TCATCAAAATACATCGCTATCCAATGTTCAAAAATTACATTATTTGAAAA   1057

genomic           1197   ccagtgttatcggtgaagcagaagggctgttgaaacatattcaagaaacc   1246
                         ||||||||||||||||||||||||||||||||||||||||||||||||||
medium            1058   CCAGTGTTATCGGTGAAGCAGAAGGGCTGTTGAAACATATTCAAGAAACC   1107

genomic           1247   gaaagtaactacgagcaggccctgttgatacttagacaaagatattgaaa   1296
                         ||||||||||||||||||||||||||||||||||||||||||||||||||
medium            1108   GAAAGTAACTACGAGCAGGCCCTGTTGATACTTAGACAAAGATATTGAAA   1157

genomic           1297   taagaggttaatcgtgaactctctcttcaaacgacttttttggacaaaaga   1346
                         ||||||||||||||||||||||||||||||||||||||||||||||||||
medium            1158   TAAGAGGTTAATCGTGAACTCTCTCTTCAAACGACTTTTTGGACAAAAGA   1207

genomic           1347   aaattgtcacgccgacgtcagggcagttgagatccctattagataccact   1396
                         ||||||||||||||||||||||||||||||||||||||||||||||||||
medium            1208   AAATTGTCACGCCGACGTCAGGGCAGTTGAGATCCCTATTAGATACCACT   1257

genomic           1397   aatgagtgcctaaataatctgaaaaacctaaatgttagcactaatgactg   1446
                         ||||||||||||||||||||||||||||||||||||||||||||||||||
medium            1258   AATGAGTGCCTAAATAATCTGAAAAACCTAAATGTTAGCACTAATGACTG   1307


genomic           1447   ggagccgattctcatatttcatgtcgtacaaaaattggacccagatacac   1496
                         ||.|||||||||||||||||||||||||||||||||||||||||||||||
medium            1308   GGGGCCGATTCTCATATTTCATGTCGTACAAAAATTGGACCCAGATACAC   1357

genomic           1497   acaaggcttgggaggaacatgcgtacaaggcggaccatgatttgctgcca   1546
                         ||||||||||||||||||||||||||||||||||||||||||||||||||
medium            1358   ACAAGGCTTGGGAGGAACATGCGTACAAGGCGGACCATGATTTGCTGCCA   1407

genomic           1547   acctgggaggagcttcgaacgttcttagaaggaaaattccgtacactcga   1596
                         ||||||||||||||||||||||||||||||||||||||||||||||||||
medium            1408   ACCTGGGAGGAGCTTCGAACGTTCTTAGAAGGAAAATTCCGTACACTCGA   1457

genomic           1597   actcatcgcaccttcgacttcagcaccacgagagaaaaaacctgtaagag   1646
                         ||||||||||||||||||||||||||||||||||||||||||||||||||
medium            1458   ACTCATCGCACCTTCGACTTCAGCACCACGAGAGAAAAAACCTGTAAGAG   1507

genomic           1647   aaaaaacattccatatcaatacaccaacttcaccgacgacgacgacgaaa   1696
                         ||||||||||||||||||||||||||||||||||||||||||||||||||
medium            1508   AAAAAACATTCCATATCAATACACCAACTTCACCGACGACGACGACGAAA   1557

genomic           1697   acctgcataatgtgtagtcaggatcatactctgtagacaatatagtaggc   1746
                         ||||||||||||||||||||||||||||||||||||||||||||||||||
medium            1558   ACCTGCATAATGTGTAGTCAGGATCATACTCTGTAGACAATATAGTAGGC   1607

genomic           1747   aaaagaaagtcctctaacgcatatgacgccacgccgagtaaggccgcgaa   1796
                         ||||||||||||||||||||
medium            1608   AAAAGAAAGTCCTCTAACGC------------------------------   1627

genomic           1797   gggagccctcgcccccctgcgctgaatgtgctcagctcaagacaagagttg   1846

medium            1628   --------------------------------------------------   1627

genomic           1847   ttgagagtaaactactcattgaacaaacaataaacgaagaagcagatcgc   1896

medium            1628   --------------------------------------------------   1627
```

```
genomic         1897 agtctgctggttaagaagaagtg   1919

medium          1628 -----------------------   1627
```

Pxyfem 6
Pxyfem 5
10bp repeat sequence
PLTR1
34bp repeat sequence

```
#=======================================
# Aligned_sequences: 2
# 1: genome.sequence.(b/w.pxyfem.6-7)
# 2: short.1200bp Band
# Matrix: EBLOSUM62
# Gap_penalty: 10.0
# Extend_penalty: 0.5
#
# Length: 1451
# Identity:    1124/1451 (77.5%)
# Similarity:  1124/1451 (77.5%)
# Gaps:         318/1451 (21.9%)
# Score: 6560.0
#=======================================

genome               1 ataatcttcttaaccagcagactgcgatctgcttcttcgtttattgtttg     50

short                1 --------------------------------------------------      0

genome              51 ttcaatgagtagtttactctcaacaactcttgtcttgagctgagcacatt    100

short                1 --------------------------------------------------      0

genome             101 cagcgcagggggcgagggctcccttcgcggccttactcggcgtggcgtca    150

short                1 --------------------------------------------------      0

genome             151 tatgcgttagaggactttcttttgcctactatattgtgaaattgtttgca    200
                                            |||||||||||||||||||||||||||||
short                1 ---------------------TTGCCTACTATATTGTGAAATTGTTTGCA     29

genome             201 gtggcacagagaatgatcctgactacacattatgcaggttttcgtcgtct    250
                       ||||||||||||||||||||||||||||||||||||||||||||||||||
short               30 GTGGCACAGAGAATGATCCTGACTACACATTATGCAGGTTTTCGTCGTCT     79

genome             251 tcgtcggtgaagttggtgtattgatatggaatgtttttctcttacaggt    300
                       ||||||||||||||||||||||||||||||||||||||||||||||||||
short               80 TCGTCGGTGAAGTTGGTGTATTGATATGGAATGTTTTTCTCTTACAGGT    129

genome             301 tttttctctcgtggtgctgaagtcgaaggtgcgatgagttcgagtgtacg    350
                       ||||||||||||||||||||||||||||||||||||||||||||||||||
short              130 TTTTTCTCTCGTGGTGCTGAAGTCGAAGGTGCGATGAGTTCGAGTGTACG    179

genome             351 gaattttccttctaagaacgttcgaagctcctcccaggttggcagcaaat    400
                       ||||||||||||||||||||||||||||||||||||||||||||||||||
short              180 GAATTTTCCTTCTAAGAACGTTCGAAGCTCCTCCCAGGTTGGCAGCAAAT    229

genome             401 catggtccgccttgtacgcatgttcctcccaagccttgtgtgtatctggg    450
                       ||||||||||||||||||||||||||||||||||||||||||||||||||
short              230 CATGGTCCGCCTTGTACGCATGTTCCTCCCAAGCCTTGTGTGTATCTGGG    279

genome             451 tccaatttttgtacgacatgaaatatgagaatcggcccccagtcattagt    500
                       ||||||||||||||||||||||||||||||||||||||||||||||||||
short              280 TCCAATTTTTGTACGACATGAAATATGAGAATCGGCCCCCAGTCATTAGT    329

genome             501 gctaacatttaggtttttcaaattatttaggcactcattagtggtatcta    550
                       |||||||||||.|||||||||||||||||.||||||||||||||||||||
```

```
short            330 GCTAACATTTACGTTTTTCAAATTATTTACGCACTCATTAGTGGTATCTA    379

genome           551 atagggatctcaactgccctgacgtcggcgtgacaattttcttttgtcca    600
                     |||.|||||||.||||||||||||||||||||||||||||||||||||||
short            380 ATACGGATCTCTACTGCCCTGACGTCGGCGTGACAATTTTCTTTTGTCCA    429

genome           601 aaaagtcgtttgaagagagagttaaatttcccattcaattccacgtcgac    650
                     ||||||||||||||||.|||||||||||||||||||||||||..||||||
short            430 AAAAGTCGTTTGAAGATAGAGTTAAATTTCCCATTCAATTCCTAGTCGAC    479

genome           651 tgttgctgcttactctctgtatgattccgtctagcgctattaggaagagc    700
                     |||.|.||||||||||||||||||||||||||||||||||||||||||||
short            480 TGTAGATGCTTACTCTCTGTATGATTCCGTCTAGCGCTATTAGGAAGAGC    529

genome           701 aagggcgacaaaagacagccctgacggatcccagcgtgcacggcgacatc    750
                     ||||||||||||||||||||||||||||||||||||||||||||||||||
short            530 AAGGGCGACAAAAGACAGCCCTGACGGATCCCAGCGTGCACGGCGACATC    579

genome           751 ctccgagattaggccaatgagagatggcctttataagatgtattatctta    800
                     ||||||||||||||||||||||||||||||||||||||||||||||||||
short            580 CTCCGAGATTAGGCCAATGAGAGATGGCCTTTATAAGATGTATTATCTTA    629

genome           801 atcgggacaccaacggataataagcgggaccacaagctgcaccatctcaa    850
                     ||||||||||||||||||||||||||||||||||||||||||||||||||
short            630 ATCGGGACACCAACGGATAATAAGCGGGACCACAAGCTGCACCATCTCAA    679

genome           851 cgtatcgaaagctttttcgaaatcgacgaaagtaaggtatacctccctct    900
                     ||||||||||||||||||||||||||||||||||||||||||||||||||
short            680 CGTATCGAAAGCTTTTTCGAAATCGACGAAAGTAAGGTATACCTCCCTCT    729

genome           901 gccattctgatgcttgttcaagtatgatacggagagtgttaatttggtag    950
                     ||||||||||||||||||||||||||||||||||||||||||||||||||
short            730 GCCATTCTGATGCTTGTTCAAGTATGATACGGAGAGTGTTAATTTGGTAG    779

genome           951 gtgcaagagcgattaaggcgaaagctttttattttctttttcgggttctg   1000
                     ||||||||||||||||||||||||||||||||||||||||||||||||||
short            780 GTGCAAGAGCGATTAAGGCGAAAGCTTTTTATTTTCTTTTTCGGGTTCTG    829

genome          1001 taagtttctcttcattattattaatgtcttctgatatagttacagtctct   1050
                     ||||||||||||||||||||||||||||||||||||||||||||||||||
short            830 TAAGTTTCTCTTCATTATTATTAATGTCTTCTGATATAGTTACAGTCTCT    879

genome          1051 gaaatgaaaattatactagtaaatccagctttatatattataatttctgc   1100
                     ||||||||||||||||||||||||||||||||||||||||||||||||||
short            880 GAAATGAAAATTATACTAGTAAATCCAGCTTTATATATTATAATTTCTGC    929

genome          1101 ttcccaacatgtaactgctggggtcaagtttggttcgcccactgggcgtg   1150
                     ||||||||||||||||||||||||||||||||||||||||||||||||||
short            930 TTCCCAACATGTAACTGCTGGGGTCAAGTTTGGTTCGCCCACTGGGCGTG    979

genome          1151 ggtctcttttttgcactacgggcgtgccctcaataaatgcatccacctccc   1200
                     ||||||||||||||||||||||||||||||||||||||||||||||||||
short            980 GGTCTCTTTTTGCACTACGGGCGTGCCCTCAATAAATGCATCCACCTCCC   1029

genome          1201 tcgccttgtttggcggcacaagcgccagaagaagctgtttcctcgtcaca   1250
                     ||||||||||||||||||||||||||||||||||||||||||||||||||
short           1030 TCGCCTTGTTTGGCGGCACAAGCGCCAGAAGAAGCTGTTTCCTCGTCACA   1079

genome          1251 taatcttccgacttcttcttaaccagcagactgcgatctgcttcttcgtt   1300
                     ||||||||||||||||||||||||||||||||||||||||||||||||||
short           1080 TAATCTTCCGACTTCTTCTTAACCAGCAGACTGCGATCTGCTTCTTCGTT   1129

genome          1301 tattgtttgttcaatgagtagtttactctcaacaactcttgtcttgagct   1350
                     ||||
short           1130 TATT----------------------------------------------   1133

genome          1351 gagcacattcagcgcaggggacgagggctcccttcgcggccttactcggc   1400
```

```
short            1134 --------------------------------------------------   1133

genome           1401 gtggcgtcatatgcgttagaggactttattttgcctactatattgtgaaa   1450

short            1134 --------------------------------------------------   1133
```

Pxyfem 6
10bp repeat
PLTR1
Pxyfem 7
34 bp repeat sequence

```
#=======================================
# Aligned_sequences: 2
# 1: genomic.sequence.(b/w.pxyfem.7-8)
# 2: largeband.2.2kb
# Matrix: EDNAFULL
# Gap_penalty: 10.0
# Extend_penalty: 0.5
#
# Length: 2404
# Identity:    2120/2404 (88.2%)
# Similarity:  2120/2404 (88.2%)
# Gaps:         261/2404 (10.9%)
# Score: 10418.0
#=======================================

genomic             1 tgtagacaatatagtaggcaaaagaaagtcctctaacgcatatgacgcca     50

largeband           1 --------------------------------------------------      0

genomic            51 cgccgagtaaggccgcgaagggagccatcgccccctgcgctgaatgtgct    100

largeband           1 --------------------------------------------------      0

genomic           101 cagctcaagacaagagttgttgagagtaaactactcattgaacaaacaat    150
                                                       ||| ||| |||||||||
largeband           1 ---------------------------------CTC-TTG-ACAAACAAT     15

genomic           151 aaacgaagaagcagatcgcagtctgctggttaagaagaagtctgctggtt    200
                      |||||||||||||||||||||||||||||||||||||||||||||||.||
largeband          16 AAACGAAGAAGCAGATCGCAGTCTGCTGGTTAAGAAGAAGTCTGCTGATT     65

genomic           201 aagcaggccctgttgatacttagacaaagatatggaaataagaggttaat    250
                      ||||..|||||||||||.||||.||.||||||..|||||||.|||||.||
largeband          66 AAGCGAGCCCTGTTGATCCTTATACGAAGATAGTGAAATAAAAGGTTGAT    115

genomic           251 cgtgaactctctcttcaaacgacttttttggacaaagaaaattgtcacgg    300
                      ||.|||||||||||||..||||||||||||||||||||.||||||||| |
largeband         116 CGGGAACTCTCTCTTCTTACGACTTTTTTGGACAAAGAGAATTGTCAC-G    164

genomic           301 ccgacgtcagggcagttgagatccctattagataccactaatgagtgcct    350
                      ||||||||.|||.||||||.||||||||||.|||||||||||||||||||
largeband         165 CCGACGTCCGGGGAGTTGACATCCCTATTACATACCACTAATGAGTGCCT    214

genomic           351 aaataatttgaaaaatctaaatgttagcactaatgactg-ggggccgatt    399
                      ||||||||||||.|||||||||||||||||||||||||| ||||||||||
largeband         215 AAATAATTTGAATAATCTAAATGTTAGCACTAATGACTGTGGGGCCGATT    264

genomic           400 ctcatatttcatgtcgtacaaaaattggacccagatacacacaaggctat    449
                      ||||||||||||||||||||||||||||||||||||||||||||||||||
largeband         265 CTCATATTTCATGTCGTACAAAAATTGGACCCAGATACACACAAGGCTAT    314

genomic           450 actataccgaagatattccggacgtccagtctagacgagattagtatggc    499
                      ||||||||||||||||||||||||| |||||||.||.||||||||||||.
largeband         315 ACTATACCGAAGATATTCCGGACGT-CAGTCTACACCAGATTAGTATGGG    363
```

```
genomic          500 tctcaaacagctaaaaaacaacaaagctccgggagatgatggaataacga    549
                     ||||||||||||||||||||||||||||||||||||||||||||||||||
largeband        364 TCTCAAACAGCTAAAAAACAACAAAGCTCCGGGAGATGATGGAATAACGA    413

genomic          550 cagaacttctggaaagccggcggtagaccgattttaatagcacttcggag    599
                     |.|||||||| |||||||||||||||||||||||||||||||||||||||
largeband        414 CGGAACTTCT-GAAAGCCGGCGGTAGACCGATTTTAATAGCACTTCGGAG    462

genomic          600 gctgttttaattcctcatactcgaaggcacaagcccggaggcatggagaa    649
                     |||| |||||||||||||||||||||||||||||||||||||||||||||
largeband        463 GCTG-TTTAATTCCTCATACTCGAAGGCACAAGCCCGGAGGCATGGAGAA    511

genomic          650 gaagtgtagtgactttgttcttcaagaaaggcaacaaagccctattgaag    699
                     ||||||||||||||||||||||||||||||||||||||||||||||||||
largeband        512 GAAGTGTAGTGACTTTGTTCTTCAAGAAAGGCAACAAAGCCCTATTGAAG    561

genomic          700 aattatagacccattgcacttctgagtcatgtgtacaagctgttttcgag    749
                     ||||||||||||||||||||||||||||||||||||||||||||||||||
largeband        562 AATTATAGACCCATTGCACTTCTGAGTCATGTGTACAAGCTGTTTTCGAG    611

genomic          750 agttattacgaatcgtctcgagcaaagactcgacgacttccagtcacccg    799
                     ||||||||||||||||||||||||||||||||||||||||||||||||||
largeband        612 AGTTATTACGAATCGTCTCGAGCAAAGACTCGACGACTTCCAGTCACCCG    661

genomic          800 aacaagccgggttccgaaaaggctatagtaccatagatcatatacacacg    849
                     ||||||||||||||||||||||||||||||||||||||||||||||||||
largeband        662 AACAAGCCGGGTTCCGAAAAGGCTATAGTACCATAGATCATATACACACG    711

genomic          850 cttcggcaggttatacagaagaccgaggagtataatctacctttatgtct    899
                     ||||||||||||||||||||||||||||||||||||||||||||||||||
largeband        712 CTTCGGCAGGTTATACAGAAGACCGAGGAGTATAATCTACCTTTATGTCT    761

genomic          900 agcgtttgtggactatgagaaagcctttgattctattgagctctgggcga    949
                     ||||||||||||||||||||||||||||||||||||||||||||||||||
largeband        762 AGCGTTTGTGGACTATGAGAAAGCCTTTGATTCTATTGAGCTCTGGGCGA    811

genomic          950 tgcttcaatcccttcagcggtgccatatagactatcgctatatcgaggtg    999
                     ||||||||||||||||||||||||||||||||||||||||||||||||||
largeband        812 TGCTTCAATCCCTTCAGCGGTGCCATATAGACTATCGCTATATCGAGGTG    861

genomic         1000 ttgagatgtatgtacaatgctgccacaatgtcagttcgattacacgaaca   1049
                     ||||||||||||||||||||||||||||||||||||||||||||||||||
largeband        862 TTGAGATGTATGTACAATGCTGCCACAATGTCAGTTCGATTACACGAACA    911

genomic         1050 tagcacaaaaccgatccagttgcaaaggggcgtgagacagggagatgtta   1099
                     ||||||||||||||||||||||||||||||||||||||||||||||||||
largeband        912 TAGCACAAAACCGATCCAGTTGCAAAGGGGCGTGAGACAGGGAGATGTTA    961

genomic         1100 tttctccgaaactgtgcactggaagatgtttttaagcttgtagagtggaa   1149
                     ||||||||||||||||||||||||||||||||||||||||||||||||||
largeband        962 TTTCTCCGAAACTGTGCACTGGAAGATGTTTTTAAGCTTGTAGAGTGGAA   1011

genomic         1150 aagactgggcattaacgtcaatggcgaatatatctctcatctacgatttg   1199
                     ||||||||||||||||||||||||||||||||||||||||||||||||||
largeband       1012 AAGACTGGGCATTAACGTCAATGGCGAATATATCTCTCATCTACGATTTG   1061

genomic         1200 ctgatgacatagttattatggcggaaacgctggaggagttaggcgaaatg   1249
                     ||||||||||||||||||||||||||||||||||||||||||||||||||
largeband       1062 CTGATGACATAGTTATTATGGCGGAAACGCTGGAGGAGTTAGGCGAAATG   1111

genomic         1250 cacacagacctcaatcatgcctctaaacaagttgggctgaaaatgaacat   1299
                     ||||||||||||||||||||||||||||||||||||||||||||||||||
largeband       1112 CACACAGACCTCAATCATGCCTCTAAACAAGTTGGGCTGAAAATGAACAT   1161

genomic         1300 ggacaagacaaaggtcatgtcgaacgaacatgtttcatcatcgcccgtaa   1349
                     ||||||||||||||||||||||||||||||||||||||||||||||||||
largeband       1162 GGACAAGACAAAGGTCATGTCGAACGAACATGTTTCATCATCGCCCGTAA   1211
```

```
genomic         1350 ctgtaggaggtgtcaccatcgaagttgtcgatcagtatccctacctagga   1399
                     ||||||||||||||||||||||||||||||||||||||||||||||||||
largeband       1212 CTGTAGGAGGTGTCACCATCGAAGTTGTCGATCAGTATCCCTACCTAGGA   1261

genomic         1400 caagtgatccgattaggtaaatccaactttgataaagaggtagctcgtag   1449
                     ||||||||||||||||||||||||||||||||||||||||||||||||||
largeband       1262 CAAGTGATCCGATTAGGTAAATCCAACTTTGATAAAGAGGTAGCTCGTAG   1311

genomic         1450 aatccaactcggatgggcagcgttcgggaaattacgacacatcttcactg   1499
                     ||||||||||||||||||||||||||||||||||||||||||||||||||
largeband       1312 AATCCAACTCGGATGGGCAGCGTTCGGGAAATTACGACACATCTTCACTG   1361

genomic         1500 aaaacatacctcagtgtcaaaaacaaaagttttcaatcagtgcgtgttgc   1549
                     ||||||||||||||||||||||||||||||||||||||||||||||||||
largeband       1362 AAAACATACCTCAGTGTCAAAAACAAAAGTTTTCAATCAGTGCGTGTTGC   1411

genomic         1550 cagtgatgacttacgaagccgagacgtggtgcttcaccaaagggcttatc   1599
                     ||||||||||||||||||||||||||||||||||||||||||||||||||
largeband       1412 CAGTGATGACTTACGAAGCCGAGACGTGGTGCTTCACCAAAGGGCTTATC   1461

genomic         1600 cacaagctcagagttgctcagcgtgctatggaaagggctatgttaggcgt   1649
                     ||||||||||||||||||||||||||||||||||||||||||||||||||
largeband       1462 CACAAGCTCAGAGTTGCTCAGCGTGCTATGGAAAGGGCTATGTTAGGCGT   1511

genomic         1650 gtccctgcgagataggattcgtaatgaagaaatccgcaggagaactaaag   1699
                     ||||||||||||||||||||||||||||||||||||||||||||||||||
largeband       1512 GTCCCTGCGAGATAGGATTCGTAATGAAGAAATCCGCAGGAGAACTAAAG   1561

genomic         1700 ttaccgacatagccaaaaggattagcacgctgaagtggcaatgggctgg-   1748
                     ||||||||||||||||||||||||||||||||||||||||||||||||| 
largeband       1562 TTACCGACATAGCCAAAAGGATTAGCACGCTGAAGTGGCAATGGGCTGGC   1611

genomic         1749 ccacgtagcccgcagagccgacgaccgctggagtacaaaggttctggagt   1798
                     ||||||||||||||||||||||||||||||||||||||||||||||||||
largeband       1612 CCACGTAGCCCGCAGAGCCGACGACCGCTGGAGTACAAAGGTTCTGGAGT   1661

genomic         1799 ggagaccccgtgtcggcaaacggcgtgtcggtcgccccccaacccgttgg   1848
                     ||||||||||||||||||||||||||||||||||||||||||||||||||
largeband       1662 GGAGACCCCGTGTCGGCAAACGGCGTGTCGGTCGCCCCCCAACCCGTTGG   1711

genomic         1849 tctgatgatctgcggaaggtagcgggaagccgctggatgcagatggcggg   1898
                     ||||||||||||||||||||||||||||||||||||||||||||||||||
largeband       1712 TCTGATGATCTGCGGAAGGTAGCGGGAAGCCGCTGGATGCAGATGGCGGG   1761

genomic         1899 tgaccgtttggggtggcgatcgttaggagaggcctatgtccaacagtgga   1948
                     ||||||||||||||||||||||||||||||||||||||||||||||||||
largeband       1762 TGACCGTTTGGGGTGGCGATCGTTAGGAGAGGCCTATGTCCAACAGTGGA   1811

genomic         1949 ctaaagaaggctgagagagagagagacacacaaggcttgggcggaccatg   1998
                     ||||||||||||||||||||||||||||||||||||||||||||||||||
largeband       1812 CTAAAGAAGGCTGAGAGAGAGAGAGACACACAAGGCTTGGGCGGACCATG   1861

genomic         1999 atttgctgccaacctgggaggagcttcgaacgttcttagaaggaaaattc   2048
                     ||||||||||||||||||||||||||||||||||||||||||||||||||
largeband       1862 ATTTGCTGCCAACCTGGGAGGAGCTTCGAACGTTCTTAGAAGGAAAATTC   1911

genomic         2049 cgtacactcgaactcatcgcaccttcgacttcagcaccacgagagaacaa   2098
                     ||||||||||||||||||||||||||||||||||||||||||||||||||
largeband       1912 CGTACACTCGAACTCATCGCACCTTCGACTTCAGCACCACGAGAGAACAA   1961

genomic         2099 acctgtaagagaaaaaacattccatatcaatacaccaacttcaccgacga   2148
                     ||||||||||||||||||||||||||||||||||||||||||||||||||
largeband       1962 ACCTGTAAGAGAAAAAACATTCCATATCAATACACCAACTTCACCGACGA   2011

genomic         2149 cgacgacgacgaaaacctgcataatgtgtagtcaggatcatactctgtgc   2198
                     ||||||||||||||||||||||||||||||||||||||||||||||||||
largeband       2012 CGACGACGACGAAAACCTGCATAATGTGTAGTCAGGATCATACTCTGTGC   2061
```

```
genomic         2199 cactgcaaacaatttcacaatatagtaggcaaaataaagtcctctaacgc   2248
                     ||||||||||||||||||||||||||||||||||||||||||||||||||
largeband       2062 CACTGCAAACAATTTCACAATATAGTAGGCAAAATAAAGTCCTCTAACGC   2111

genomic         2249 atatgacgccacgccgagtaaggccgcg-aagggagccctcgtcccctgc   2297
                     |||||||||||||||||||||||||||| ||||||
largeband       2112 ATATGACGCCACGCCGAGTAAGGCCGCGAAAGGGA---------------   2146

genomic         2298 gctgaatgtgctcagctcaagacaagagttgttgagagtaaactactcat   2347

largeband       2147 --------------------------------------------------   2146

genomic         2348 tgaacaaacaataaacgaagaagcagatcgcagtctgctggttaagaaga   2397

largeband       2147 --------------------------------------------------   2146

genomic         2398 agtc    2401

largeband       2147 ----    2146
```

pxyfem 8
PLTR2
10bp Repeat
PLTR1
pxyfem 7

Figure S3: **Sequencing and analysis of three gDNA + bands from figure 2B.** Sanger sequencing of bands extracted from gel in Figure 2B (positive genomic control). Sequences were aligned to CABWKK010000004 genomic scaffold and identified as the intervening genomic sequences between *Pxyfem* loci 5-6 (medium sized band), 6-7 (short sized band) and 7-8 (large sized band). These four *Pxyfem* loci occur in the second genomic cluster where the loci are more closely clustered together, possibly facilitating PCR between these loci. Identified areas are coloured in the clustal alignments and labelled below each alignment, accordingly.

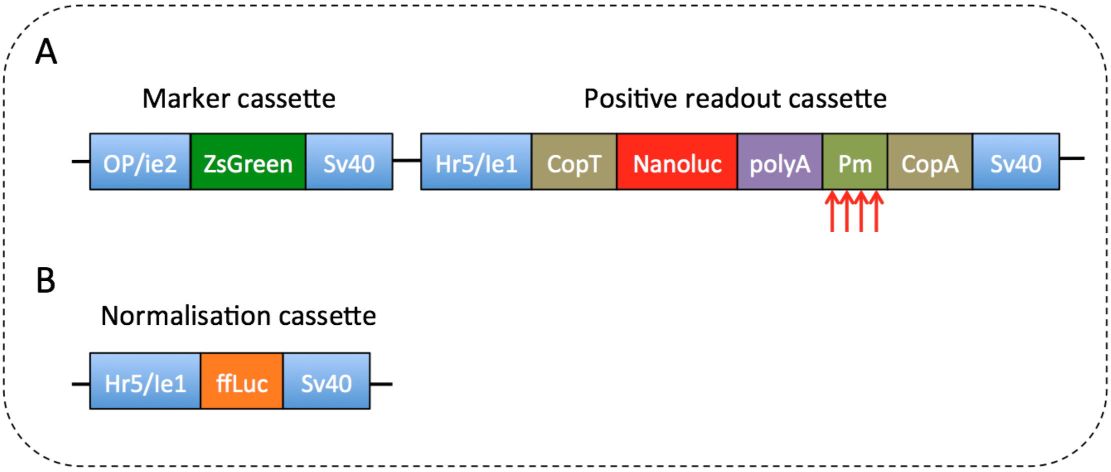

Figure S4: **Constructs used for positive readout cleavage experiment.** A) Schematic of AGG2208. In the absence of transcript cleavage, the bacterial hairpins CopT and CopA dampen translation of the positive

readout mRNA transcript. In the presence of transcript cleavage (signified by the red arrows) the CopA inhibitory hairpin is removed and the internal, synthetic 120bp polyA tail exposed, allowing increased translation of the transcript and higher nanoluciferase signal to be observed. 'Pm' represents the 325bp sequence of *PxyMasc* against which *Pxyfem* matches (i.e. the putative *PxyMasc* target site of *Pxyfem*). B) Schematic of AGG1186. 'ffLuc' represents the firefly luciferae ORF.

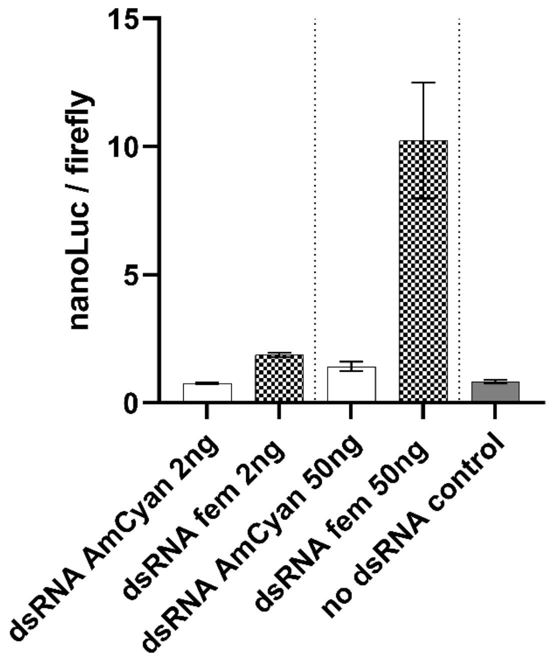

Figure S5: **Results of initial testing of AGG2208 in Aag2 cells.** Normalised luciferase values (nLuc/firefly) for transfection experiments with dsRNA targeting the 325bp *PxyMasc* sequence in AGG2208 (treatment – 'dsRNA fem') or a 456bp sequence of *AmCyan* (negative control) at 2ng or 50ng per well, or without co-transfection of any dsRNA. Agg2208 and AGG1186 were co-transfected into each well. Means are given for 8 biological replicates.
